# Performance Evaluation of Geometrically Different Pediatric Arterial Cannulae in a Pediatric Cardiopulmonary Bypass Model

**DOI:** 10.21470/1678-9741-2023-0110

**Published:** 2023-11-08

**Authors:** Gabriela B. de O. Carvalho, Luiz Fernando Caneo, Gregory Matte, Caio Henrique de A. Cruz, Everton Neri da Silva, Luciana P. Carletto, Ana Vitória C. X. de Castro, Betina G. Madueño Silva, Valéria C. Policarpo, Idágene A. Cestari, Fabio B Jatene, Marcelo Biscegli Jatene

**Affiliations:** 1 Cardiovascular Surgery Division, Instituto do Coração, Faculdade de Medicina, Universidade de São Paulo, São Paulo, São Paulo, Brazil; 2 Department of Cardiac Surgery, Boston Children’s Hospital, Boston, Massachusetts, United States of America

**Keywords:** Congenital Heart Disease, Cardiopulmonary Bypass, Cannula, Hematocrit, Ringer’s Lactate, Heart-Lung Machine

## Abstract

**Objective:**

To define a reference chart comparing pressure drop vs. flow generated by a set of arterial cannulae currently utilized in cardiopulmonary bypass conditions in pediatric surgery.

**Methods:**

Cannulae from two manufacturers were selected considering their design and outer and inner diameters. Cannula performance was evaluated in terms of pressure drop vs. flow during simulated cardiopulmonary bypass conditions. The experimental circuits consisted of a Jostra HL-20 roller pump, a Quadrox-i pediatric oxygenator (Maquet Cardiopulmonary AG, Rastatt, Germany), and a custom pediatric tubing set. The circuit was primed with lactated Ringer’s solution only (first condition) and with human packed red blood cells added (second condition) to achieve a hematocrit of 30%. Cannula sizes 8 to 16 Fr were inserted into the cardiopulmonary bypass circuit with a “Y” connector. The flow was adjusted in 100 ml/min increments within typical flow ranges for each cannula. Pre-cannula and post-cannula pressures were measured to calculate the pressure drop.

**Results:**

Utilizing a pressure drop limit of 100 mmHg, our results suggest a recommended flow limit of 500, 900, 1400, 2600, and 3100 mL/min for Braile arterial cannulae sizes 8, 10, 12, 14, and 16 Fr, respectively. For Medtronic DLP arterial cannulae sizes 8, 10, 12, 14, and 16 Fr, the recommended flow limit is 600, 1100, 1700, 2700, and 3300 mL/min, respectively.

**Conclusion:**

This study reinforces discrepancies in pressure drop between cannulae of the same diameter supplied by different manufacturers and the importance of independent translational research to evaluate components’ performance.

## INTRODUCTION

**Table:** 

Abbreviations, Acronyms & Symbols	
BSA	= Body surface area
CPB	= Cardiopulmonary bypass

Perfusion practice during cardiovascular surgery is recognized in the international literature as a critical component for successful patient outcomes. Patients with congenital heart disease requiring cardiopulmonary bypass (CPB) during their surgical repair demand a specific bypass plan starting with the basics of patient’s bodyweight and body surface area (BSA), anticipated pump flow requirements, allergy history, original diagnosis, previous surgeries, and current indications for surgery^[1]^.

The perfusionist must select and assemble an array of devices and equipment matched to the patient’s size, expected pump flow rates, and other factors related to diagnosis; also, the perfusionist must choose the most suitable CPB components to minimize adverse effects of hemolysis, pressure drop, resistance, hemodilution, etc. Circuit components including the pump, oxygenator, arterial filter, and arterial cannula have a collective impact on the amount of beneficial hemodynamic energy delivered to the patient. The ascending aorta is most commonly cannulated for arterial inflow. The arterial cannula, whose diameter must be compatible with the aorta’s size, is usually the component with the greatest resistance to flow in the CPB circuit, especially in neonates^[2]^. Hence, the arterial cannula’s size should be chosen with caution according to the flow needs of each patient^[3]^. Smaller arterial cannulae may lead to higher jet velocities, higher shear stress, and higher pressure drop across the cannula, depending on the flow rates used^[3]^. These factors, as well as hypothermia, longer CPB duration, exposure to significant foreign surface area, and ischemia-reperfusion injury, may further increase red blood cell damage and platelet activation during CPB procedures, which may be associated with postoperative mortality and morbidity^[1]^.

Several pediatric aortic cannulae are available for clinical use with noticeable differences in geometry, inner diameter, outer diameter, pressure drops, and hemodynamic energy delivery capabilities despite being labeled with similar sizes^[4]^.

The overall performance data for cannulae provided by the manufacturers is based on tests using water as the perfusate fluid. The ideal test would utilize blood as the perfusate. Therefore, acquiring the best available information requires testing with human blood and pre-established hematocrit and temperature ranges. This fact was demonstrated by Undar et al.^[4]^ when they showed experimentally that same-sized pediatric arterial cannulae currently on the market exhibited significant performance differences.

Brazil has many medical devices approved by the Agência Nacional de Vigilância Sanitária (or ANVISA, the National Health Surveillance Agency), manufactured and available for domestic use only. Some devices are commercialized with no sufficient clinical data or benchmarking with similar devices^[5]^. Therefore, it is not surprising that the large clinical trials published by the international scientific community are generated using products approved by the United States Food and Drug Administration (or FDA). Products available only in select markets such as Brazil are commonly compared against this data, but this must be done with caution.

Our aim was to create charts which would support the choice of domestic and internationally available pediatric cannulae to be used during CPB. In this work, we describe a simulated CPB circuit with measurements of flow *vs.* pressure drop (also called pressure loss) for conditions found in our practice. Specifically, we compared pressure drops, inner and outer diameters, and the geometric design of 8 Fr, 10 Fr, 12 Fr, 14 Fr, and 16 Fr pediatric arterial cannulae from the Brazilian manufacturer Braile Biomédica (Sao José do Rio Preto, São Paulo, Brazil) *vs.* those from Medtronic Inc. (Minneapolis, Minnesota, United States of America) in a simulated CPB circuit. Further, we developed a graph with pressure curves for the Braile cannulae.

## METHODS

### Experimental Circuits

**Fig. 1:**
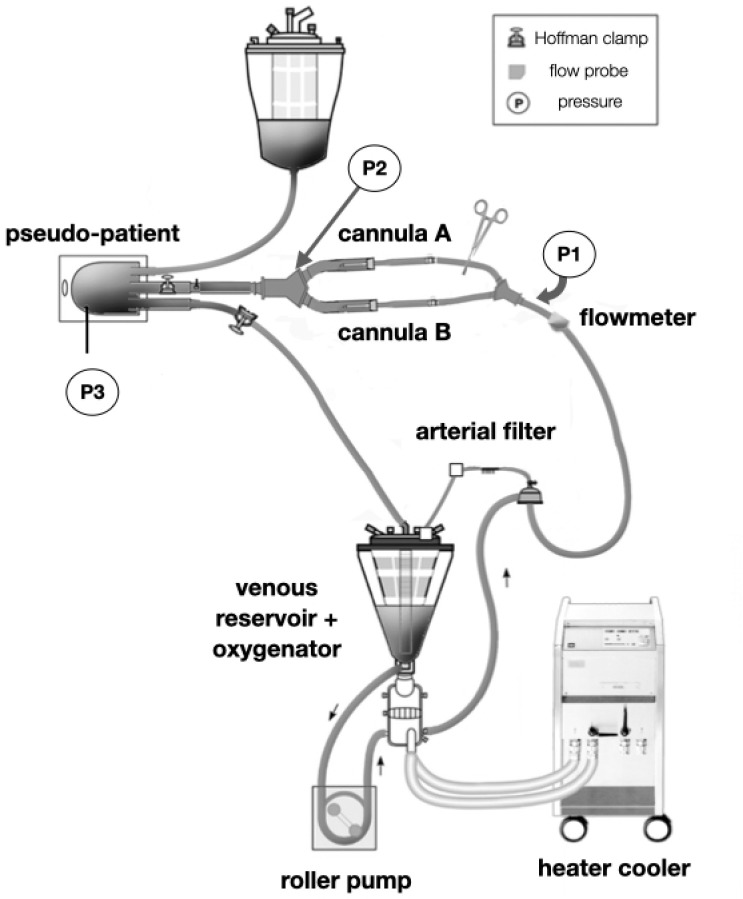
Simulated pediatric cardiopulmonary bypass circuit for arterial cannula testing.

The circuit design employed in this study simulated pediatric CPB. The mock patient and circuit consisted of a Maquet HL-20 roller pump (Maquet Cardiopulmonary AG, Rastatt, Germany), a pediatric Quadrox-iD oxygenator (Maquet Cardiopulmonary AG, Rastatt, Germany), a 1/4" × 150 cm tube as the arterial line, a 3/8" × 140 cm tube as the venous line, and a ¼" boot line connecting the outlet of venous reservoir to the inlet of the oxygenator and going through the arterial pump head, as shown in Figure 1.

A 1600 ml soft bag venous reservoir (Medtronic, Inc., Minneapolis, Minnesota, United States of America) connected to the CPB circuit was used as a pseudo-patient. The pseudo-patient was located 80 cm above the venous reservoir. One Hoffman clamp was placed after the arterial cannula insertion site to maintain a given post-cannula pressure during all trials. Another Hoffman clamp was placed on the venous line near the pseudo-patient to maintain a balance between the arterial flow rate and the venous drainage. The blood temperature was maintained by a HCU 20 heater-cooler unit (Maquet Cardiopulmonary AG, Rastatt, Germany). The CPB circuit was first primed with lactated Ringer’s solution for de-airing and then tests were performed (first condition). Following that, human-packed red blood cells were added to the circuit to maintain the blood hematocrit at 30% for further testing (second condition).

### Experimental Design

The pseudo-patient pressure (P1) was held constant at 0-2 mmHg using a Hoffman clamp placed before the venous line (P3). Post-cannula pressure (P2) was maintained at 50 mmHg during all trials using another Hoffman clamp after the arterial cannula.

Before testing, the inner and outer diameters of the cannulae were measured with a mechanical caliper (Table 1). The circuit was filled with lactated Ringer’s solution for the first set of tests. Two 8 Fr cannulae (one of each manufacturer) were inserted into the circuit using a “Y” connector. Flows from 100 to 1100 ml/min were used, and pre-cannula and post-cannula pressures were recorded, alternating a clamp on the arms of the arterial line to determine the pressure drop of each cannula. During testing, the venous reservoir volume level was kept at 200 mL.

**Table 1:** Measurements of aortic cannula diameters.

Aortic cannulae	Outer diameter (mm)	Inner diameter (mm)
8 Fr Braile	3.02	1.64
8 Fr Medtronic	2.94	1.79
10 Fr Braile	3.58	2.40
10 Fr Medtronic	3.64	2.32
12 Fr Braile	4.25	3.00
12 Fr Medtronic	4.33	2.92
14 Fr Braile	5.03	4.04
14 Fr Medtronic	5.02	4.01
16 Fr Braile	5.60	4.50
16 Fr Medtronic	5.60	4.60

Measurements were repeated 15 times for each cannula size consecutively. For each of the eight cannulae evaluated, the post-cannula pressure (mean arterial pressure of the pseudo-patient) was set at 50 mmHg using a Hoffman clamp, and the mean circuit pressure was monitored at the pre-cannula site. Due to the specifications of each cannula, the flow increases were not the same in all experiments. Flow ranges in the study were 100 to 1100 ml/min for the 8 Fr cannula, 100 to 1500 ml/min for the 10 Fr cannula, 800 to 2000 for the 12 Fr cannula, 1000 to 3000 ml/min for the 14 Fr cannula, and 2000 to 3500 ml/min for the 16 Fr cannula. In the second set of conditions, human-packed red blood cells were added into the circuit to maintain the blood hematocrit at 30% at 36.5°C. A total of 188 trials were performed, 94 with lactated Ringer’s solution only and 94 with human blood and lactated Ringer solution mixed for a hematocrit of 30%.

### Signal Recording

Flow measurement was performed with the flow probe (Transonic Systems, Inc., Ithaca, New York, United States of America) at the oxygenator outlet. Three Edwards TruWave disposable pressure transducers (Edwards Lifesciences Corp., Irvine, California, United States of America) were positioned between the oxygenator outlet and the arterial cannulae (P1), at the post-cannulae site (P2), and at the pseudo-patient (soft bag) pressure (P3). Pressure transducers were connected to pressure monitors CPB-100 (Bioengineering Division, InCor-HC-FMUSP, São Paulo, Brazil). Pressure and flowmeter outputs were connected to a DataQ DI-710 data acquisition device (DataQ, Akron, Ohio, United States of America) and then connected to a computer via universal serial bus (or USB) port. WinDaq data acquisitions software (DataQ, Akron, Ohio, United States of America) was used to record real-time data at 1000 samples per second per channel. A 30-second segment of pressure and flow was recorded for all sets of parameters.

### Statistical Analysis

Data was presented for mean and standard deviation. One-way ANOVA-repeated measures was used to compare the flows between cannulae of the same size. Tukey’s multiple comparisons test was done to identify the difference between the variables studied. Tests were considered of statistical significance if *P*-values were ≤ 0.05. All analyses were performed using IBM Corp. Released 2010, IBM SPSS Statistics for Windows, version 19.0, Armonk, NY: IBM Corp. software and GraphPad Prism software (San Diego, California, United States of America) for Mac version 6.0 (Microsoft Corporation, Redmond, Washington, United States of America).

## RESULTS

The Braile models generated significantly higher mean circuit pressures than the Medtronic cannulae tested. Details are presented in Tables 2 to 6 for each cannula size for both first and second sets of conditions.

**Table 2:** Eight-French cannulae pressure drop results (mmHg).

Flow rate (ml/min)	First condition (clear prime)		Second condition (blood)	
	Braile	Medtronic	Braile	Medtronic
100	5.69 + 0.15	4.72 + 0.14	11.74 + 0.18	9.96 + 0.13
200	16.36 + 0.12	11.36 + 0.12	26.32 + 0.18	23.71 + 0.12
300	29.76 + 0.13	20.44 + 0.08	45.87 + 0.13	38.76 + 0.14
400	46.58 + 0.10	31.95 + 0.15	71.67 + 0.08	59.04 + 0.09
500	63.39 + 0.09	46.53 + 0.09	98.08 + 0.10	87.61 + 0.07
600	85.97 + 0.12	60.32 + 0.10	122.85 + 0.15	109.77 + 0.10
700	113.82 + 0.08	77.91 + 0.12	152.79 + 0.14	135.97 + 0.13
800	147.14 + 0.11	97.21 + 0.11	184.09 + 0.12	169.34 + 0.09
900	185.36 + 0.08	126.16 + 0.15	223.59 + 0.16	201.19 + 0.08
1000	221.30 + 0.09	154.93 + 0.08	268.46 + 0.09	238.91 + 0.11
1100	265.38 + 0.08	186.77 + 0.09	315.48 + 0.12	277.36 + 0.08

**Table 6:** Sixteen-French cannulae pressure drop results (mmHg).

Flow rate (ml/min)	First condition (clear prime)		Second condition (blood)	
	Braile	Medtronic	Braile	Medtronic
2000	43.21 + 0.14	41.11 + 0.10	48.42 + 0.12	44.85 + 0.10
2100	47.83 + 0.10	45.64 +0.13	53.03 + 0.10	48.89 + 0.11
2200	51.85 + 0.11	49.16 + 0.10	55.45 + 0.14	52.44 + 0.14
2300	56.38 + 0.12	53.82 + 0.11	60.65 + 0.12	57.02 + 0.10
2400	61.42 + 0.10	58.68 +0.13	64.95 + 0.10	61.08 + 0.11
2500	66.73 + 0.11	63.58 + 0.10	69.13 + 0.12	65.16 + 0.10
2600	72.01 + 0.10	68.22 + 0.10	74.12 +0.13	69.66 + 0.10
2700	77.33 + 0.10	72.96 +0.13	79.73 + 0.10	74.56 + 0.10
2800	82.83 + 0.11	78.15 + 0.11	84.11 + 0.11	78.65 + 0.11
2900	88.69 +0.13	84.03 + 0.10	90.04 + 0.11	84.02 + 0.11
3000	94.81 + 0.10	89.53 + 0.11	95.47 + 0.10	88.95 +0.13
3100	100.56 + 0.10	95.42 + 0.11	102.11 + 0.14	94.97 + 0.12
3200	106.55 + 0.14	101.34 + 0.10	107.3 + 0.10	99.57 + 0.14
3300	112.28 + 0.10	108.11 + 0.11	113.77 + 0.14	105.11 + 0.11
3400	119.08 + 0.10	114.53 + 0.14	119.26 + 0.11	110.32 + 0.14
3500	125.67 + 0.14	121.58 + 0.11	126.45 + 0.10	117.16 + 0.10

### Pressure Drop

**Fig. 2:**
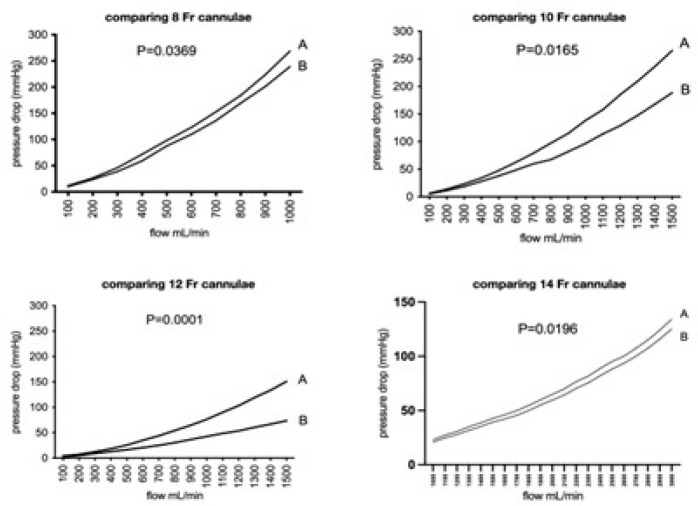
Comparison of the four pressure loss charts obtained with blood at 36°C. Charts compare flow (X axis) vs. pressure drop (Y axis) for Braile (A) and Medtronic (B) cannulae in four different sizes (8, 10, 12, and 14 Fr).

Increasing the flow rate resulted in increased pressure drops under all conditions for all cannulae, as expected. Pressure drops were observed to be higher during the second condition (with blood added to the circuit) at all flow rates when compared with the first condition (only lactated Ringer’s solution). Pressure drops in the Braile models were higher than those of the other cannulae evaluated, regardless of the condition of the experiment (Figure 2).

### Flow-Pressure Curves

**Fig. 3:**
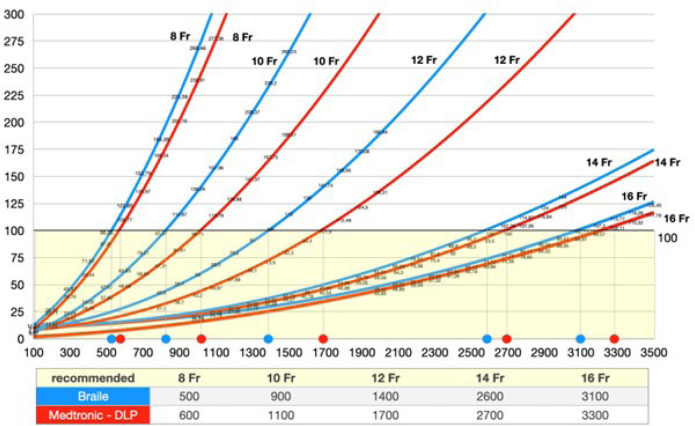
Flow-pressure curves for tested Braile and Medtronic arterial cannulae.

Figure 3 shows the flow-pressure curves of cannulae obtained using blood-primed circuits for the tested cannulae which are all available in the Brazilian market. We can see the difference between the cannulae of the same size from two different manufacturers. The maximum flow recommended for each cannula is shown in the table below the graphic based on a maximum pressure loss of 100 mmHg.

## DISCUSSION

Our results demonstrate differences in pressure drop obtained in cannulae of similar French sizing supplied by different manufacturers. Similar findings have been reported in the literature^[2-4]^. The arterial cannulae tested in this study are commercially available in Brazil and were found to vary considerably in terms of geometry, inner diameter, and pressure drops, despite being classified with the same size in French (8, 10, 12, 14, and 16 Fr). It is known that 1 Fr equals an external diameter of 0.33 mm. However, the internal diameter may differ significantly between manufacturers^[4]^.

Usually, the selection of cannula size is based on vessel diameter, cannula specifications, and the anticipated pump flow rate which is calculated based on the patient’s weight and/or BSA. Despite being more easily inserted into the aorta, smaller cannulae have increased pressure drop at a given flow and, consequently, this increases the risk to the formed elements of the blood and, potentially, the arterial wall. On the other hand, larger cannulae allow for higher flow rates with a lower pressure drop but present more risks for local vascular trauma^[7]^. Furthermore, an experienced surgeon understands the importance of appropriate perfusion management, ensuring that the flow rates and pressures are well-controlled and within safe limits. They can adapt their surgical approach and techniques to mitigate potential risks associated with larger cannulae, such as using gentle maneuvers and monitoring the cannula’s position to prevent unnecessary tissue trauma.

The pressure *vs.* flow curve is generally used as an essential guide for assessing the hemodynamic characteristics of the cannula by the industry and perfusionists. It is generally accepted that pressure drop across an arterial cannula should be limited to 100 mmHg to prevent excessive jetting, shear forces, and damage to the formed elements of the blood^[1]^.

It is an underappreciated fact that cannulae do not come from the manufacturer with rated flow rates or recommended patient sizes for their use. Instead, cannula come with charts depicting flow *vs.* pressure loss. In this study, we examined the differences in eight pediatric cannulae (sizes 8, 10, 12, 14, and 16 Fr) from two different manufacturers to determine which design elements are best suited for optimal perfusion in a simulated patient. We analyzed each cannula’s performance under different flow rates. Statistically, significant results were found in comparing same size cannulae from different manufacturers with blood having a higher pressure drops than Ringer’s lactate. This finding can be explained by the higher blood viscosity in relation to Ringer’s lactate solution^[9]^. The pressure drop variations demonstrated between the cannulae of the same French can be explained by the variation in each cannula’s length, internal diameter, and geometric design^[3,9,10]^. A thicker tubing wall in an arterial cannula is less likely to be kinked and better tolerates tubing clamps. But the added thickness decreases the internal lumen and thus decreases performance for a given external diameter. A thinner tubing wall in an arterial cannula increases the performance characteristics but also poses an increased risk of kinking at the surgical field. Arterial cannula tip style and bevel angle may also impact performance. These geometry differences are considered by the surgeon as they impact arterial cannulation during routine and emergency cannulations.

It is important to emphasize that the temperature is inversely proportional to the viscosity of the fluid, so that the lower the temperature, the greater the viscosity. In clinical practice, it is important for clinicians to recognize that a decrease in perfusate temperature results in an increased pressure drop^[2]^. We did not measure temperature as a variable in our experiment.

It would be clinically beneficial for manufacturers of pediatric cannulae to publish all relevant geometry data for their cannulae, including the inner diameter along with addition the outer diameter measurements.

## CONCLUSION

In summary, examining the performance differences among these eight cannulae allowed us to create flow recommendations for Braile and Medtronic pediatric arterial cannulae. The chart we developed may aid the perfusionist and surgeon in formulating a bypass plan for the safe conduct of CPB with objective and relevant data. Future studies may include using different priming solutions, experimental designs, and temperature ranges.

## Figures and Tables

**Table 3:** Ten-French cannulae pressure drop results (mmHg).

Flow rate (ml/min)	First condition (clear prime)		Second condition (blood)	
	Braile	Medtronic	Braile	Medtronic
100	2.68 + 0.23	2.36 + 0.18	6.44 + 0.20	5.02 + 0.19
200	6.23 + 0.14	5.82 + 0.14	13.97 + 0.15	11.25 + 0.18
300	12.77 + 0.15	10.39 + 0.15	23.28 + 0.13	18.55 + 0.09
400	21.18 + 0.19	17.11 + 0.08	34.02 + 0.17	28.04 + 0.16
500	29.59 + 0.11	23.02 + 0.17	47.67 + 0.18	37.86 + 0.10
600	40.67 + 0.08	31.14 + 0.09	62.83 + 0.10	48.68 + 0.12
700	52.28 + 0.15	40.55 + 0.14	79.21 + 0.14	59.94 + 0.08
800	65.84 + 0.17	49.43 + 0.10	97.37 + 0.09	67.31 + 0.14
900	76.28 + 0.10	61.81 + 0.13	114.87 + 0.14	81.81 + 0.10
1000	96.35 + 0.14	74.70 + 0.08	138.29 + 0.08	96.71 + 0.13
1100	113.38 + 0.09	87.33 + 0.10	157.96 + 0.13	113.79 + 0.15
1200	137.68 + 0.08	102.49 + 0.12	185.00 + 0.11	128.88 + 0.13
1300	161.83 + 0.12	118.27 + 0.13	209.37 + 0.09	147.37 + 0.08
1400	188.74 + 0.11	134.35 + 0.08	236.20 + 0.10	167.75 + 0.12
1500	214.52 + 0.14	155.80 + 0.10	265.23 + 0.08	188.51 + 0.10

**Table 4:** Twelve-French cannulae pressure drop results (mmHg).

Flow rate (ml/min)	First condition (clear prime)		Second condition (blood)	
	Braile	Medtronic	Braile	Medtronic
800	29.00 + 0.11	20.41 + 0.10	42.50 + 0.14	31.10 + 0.16
900	37.40 + 0.15	25.70 + 0.10	50.70 + 0.11	36.70 + 0.14
1000	43.20 + 0.15	30.00 + 0.12	59.00 + 0.12	43.20 + 0.15
1100	54,30 + 0.14	37.40 + 0.11	68.50 + 0.13	49.97 + 0.12
1200	63.00 + 0.17	43.60 + 0.13	79.20 + 0.18	57.68 + 0.11
1300	71.48 + 0.10	49.59 + 0.15	91.00 + 0.15	66.10 + 0.11
1400	83.15 + 0.12	58.11 + 0.14	101.00 + 0.16	73.60 + 0.10
1500	93.60 + 0.09	66.40 + 0.11	115.00 + 0.13	82.50 + 0.12
1600	110.00 + 0.09	76.30 + 0.09	130.00 + 0.09	93.40 + 0.09
1700	124.00 + 0.09	85.40 + 0.09	141.74 + 0.09	101.90 + 0.09
1800	139.00 + 0.09	94.90 + 0.09	155.65 + 0.09	112.49 + 0.09
1900	157.00 + 0.09	108.00 + 0.09	173.08 + 0.09	124.50 + 0.09
2000	171.36 + 0.09	117.34 + 0.09	190.94 + 0.09	138.31 + 0.09

**Table 5:** Fourteen-French cannulae pressure drop results (mmHg).

Flow rate (ml/min)	First condition (clear prime)		Second condition (blood)	
	Braile	Medtronic	Braile	Medtronic
1000	16.27 + 0.28	15.70 + 0.16	22.89 + 0.19	20.79 + 0.21
1100	19.08 + 0.23	17.28 + 0.14	27.29 + 0.20	24.78 + 0.20
1200	22.09 + 0.25	20.13 + 0.18	30.56 + 0.23	27.85 + 0.18
1300	25.68 + 0.22	23.56 + 0.16	34.98 + 0.18	31.72 + 0.20
1400	29.38 + 0.19	27.12 + 0.19	38.59 + 0.15	35.28 + 0.19
1500	33.04 + 0.20	30.33 + 0.18	42.74 + 0.19	39.27 + 0.17
1600	36.83 + 0.18	33.80 + 0.14	46.00 + 0.13	42.18 + 0.14
1700	40.60 + 0.15	37.22 + 0.15	49.85 + 0.12	45.54 + 0.15
1800	44.63 + 0.14	41.17 + 0.12	54.55 + 0.14	49.88 + 0.18
1900	48.62 + 0.18	45.14 + 0.13	59.79 + 0.11	55.05 + 0.12
2000	53.31 + 0.17	49.13 + 0.13	64.81 + 0.12	59.56 + 0.15
2100	58.53 + 0.15	54.36 + 0.13	69.93 + 0.12	64.30 + 0.13
2200	64.20 + 0.12	59.71 + 0.13	76.64 + 0.12	70.38 + 0.13
2300	69.60 + 0.15	64.40 + 0.13	81.50 + 0.12	75.40 + 0.11
2400	77.20 + 0.12	71.10 + 0.13	88.80 + 0.12	82.00 + 0.11
2500	83.20 + 0.10	76.90 + 0.13	95.20 + 0.12	88.20 + 0.11
2600	90.30 + 0.10	83.60 + 0.13	100.00 + 0.12	93.50 + 0.13
2700	95.88 + 0.11	91.00 + 0.13	107.26 + 0.19	100.00 + 0.13
2800	103.47 + 0.11	96.11 + 0.13	114.97 + 0.20	107.26 + 0.11
2900	111.00 + 0.11	103.07 + 0.13	124.00 + 0.23	115.84 + 0.11
3000	118.00 + 0.11	111.00 + 0.13	134.00 + 0.12	125.00 + 0.11

## References

[1] Matte GS. (2015). Perfusion for congenital heart surgery: notes on cardiopulmonary bypass
for a complex patient population.

[2] Wang S, Palanzo D, Kunselman AR, Ündar A. (2016). In vitro hemodynamic evaluation of five 6 Fr and 8 Fr arterial
cannulae in simulated neonatal cardiopulmonary bypass
circuits. Artif Organs.

[3] Rider AR, Ji B, Kunselman AR, Weiss WJ, Myers JL, Undar A. (2008). A performance evaluation of eight geometrically different 10 Fr
pediatric arterial cannulae under pulsatile and nonpulsatile perfusion
conditions in an infant cardiopulmonary bypass model. ASAIO J.

[4] Undar A, Ji B, Rider A, Lukic B, Kunselman AR, Weiss WJ (2007). Comparison of four different pediatric 10F aortic cannulae during
pulsatile versus nonpulsatile perfusion in a simulated neonatal model of
cardiopulmonary bypass. ASAIO J.

[5] Wang S, Caneo LF, Jatene MB, Jatene FB, Cestari IA, Kunselman AR (2017). In vitro evaluation of pediatric hollow-fiber membrane
oxygenators on hemodynamic performance and gaseous microemboli handling: an
international multicenter/multidisciplinary approach. Artif Organs.

[6] Ündar A. (2018). Translational research on evaluation of pediatric cardiopulmonary
bypass oxygenators. Artif Organs.

[7] Jayaraman AL, Cormican D, Shah P, Ramakrishna H. (2017). Cannulation strategies in adult veno-arterial and veno-venous
extracorporeal membrane oxygenation: techniques, limitations, and special
considerations. Ann Card Anaesth.

[8] Wang S, Force M, Kunselman AR, Palanzo D, Brehm C, Ündar A. (2019). Hemodynamic evaluation of avalon elite bi-caval dual lumen
cannulas and femoral arterial cannulas. Artif Organs.

[9] Broman LM, Prahl Wittberg L, Westlund CJ, Gilbers M, Perry da Câmara L, Westin J (2019). Pressure and flow properties of cannulae for extracorporeal
membrane oxygenation II: drainage (venous) cannulae. Perfusion.

[10] Wang S, Rosenthal T, Kunselman AR, Ündar A. (2015). Evaluation of different diameter arterial tubing and arterial
cannulae in simulated neonatal/pediatric cardiopulmonary bypass
circuits. Artif Organs.

